# Analysis of Racial/Ethnic Representation in Select Basic and Applied Cancer Research Studies

**DOI:** 10.1038/s41598-018-32264-x

**Published:** 2018-09-18

**Authors:** Santiago Guerrero, Andrés López-Cortés, Alberto Indacochea, Jennyfer M. García-Cárdenas, Ana Karina Zambrano, Alejandro Cabrera-Andrade, Patricia Guevara-Ramírez, Diana Abigail González, Paola E. Leone, César Paz-y-Miño

**Affiliations:** 1 Centro de Investigación Genética y Genómica, Facultad de Ciencias de la Salud Eugenio Espejo, Universidad UTE, Av. Mariscal Sucre and Mariana de Jesús, Block I, 2nd floor, 170129 Quito, Ecuador; 2 Gene Regulation, Stem Cells and Cancer Programme, Centre for Genomic Regulation (CRG), The Barcelona Institute of Science and Technology, Barcelona, Spain; 3 Oncology and Molecular Pathology Research Group-VHIR- Vall d’ Hebron Institut de Recerca-Vall d’ Hebron Hospital, P/de la Vall d’Hebron, Barcelona, Spain; 4 Carrera de Enfermería, Facultad de Ciencias de la Salud, Universidad de las Américas, Avenue de los Granados, Quito, 170125 Ecuador; 5 Grupo de Bio-Quimioinformática, Universidad de las Américas, Avenue de los Granados, Quito, 170125 Ecuador

## Abstract

Over the past decades, consistent studies have shown that race/ethnicity have a great impact on cancer incidence, survival, drug response, molecular pathways and epigenetics. Despite the influence of race/ethnicity in cancer outcomes and its impact in health care quality, a comprehensive understanding of racial/ethnic inclusion in oncological research has never been addressed. We therefore explored the racial/ethnic composition of samples/individuals included in fundamental (patient-derived oncological models, biobanks and genomics) and applied cancer research studies (clinical trials). Regarding patient-derived oncological models (n = 794), 48.3% have no records on their donor’s race/ethnicity, the rest were isolated from White (37.5%), Asian (10%), African American (3.8%) and Hispanic (0.4%) donors. Biobanks (n = 8,293) hold specimens from unknown (24.56%), White (59.03%), African American (11.05%), Asian (4.12%) and other individuals (1.24%). Genomic projects (n = 6,765,447) include samples from unknown (0.6%), White (91.1%), Asian (5.6%), African American (1.7%), Hispanic (0.5%) and other populations (0.5%). Concerning clinical trials (n = 89,212), no racial/ethnic registries were found in 66.95% of participants, and records were mainly obtained from Whites (25.94%), Asians (4.97%), African Americans (1.08%), Hispanics (0.16%) and other minorities (0.9%). Thus, two tendencies were observed across oncological studies: lack of racial/ethnic information and overrepresentation of Caucasian/White samples/individuals. These results clearly indicate a need to diversify oncological studies to other populations along with novel strategies to enhanced race/ethnicity data recording and reporting.

## Introduction

Oncological research encompasses many aspects which can be categorized into fundamental and applied research. Fundamental research intends to understand the principles of cancer biology. This has been studied using tumor samples, which have been used to develop patient-derived oncological models (cell lines and xenografts)^1^, biobanks^2^ and genomic-based molecular classifications^3^. The knowledge acquired in basic research has been exploited for anti-cancer drug development and further clinical trials.

Cancer cell lines have historically been used as an essential tool for the investigation of cancer-related biological processes. They also have been used for anticancer drug testing over the past 50 years^4,5^. For instance, the United States (U.S.) National Cancer Institute 60 human tumor cell lines (NCI-60) panel, developed in the late 1980s, has been exploited to study ~400,000 compounds^5^. Since then, hundreds of cancer cell lines have been developed to study the molecular basis of cancer^1^. Nowadays, the NCI has decided to refocus its drug screening strategy on patient-derived models, which maintain a more realistic tumor-stroma enviroment. The NCI Patient-Derived Models Repository (PDMR) includes patient-derived xenografts (PDXs) and *in vitro* patient-derived cell cultures (PDCs)^6^.

Another important aspect of basic research is the identification of tumor biomarkers, which can be used for early cancer detection, diagnosis and prognosis. Cancer biomarkes are usually studied using body liquid biopsies or tissue samples deposited in biobanks^2,7,8^. One of these biobanks has been developed by the NCI Tissue Array Research Program (TARP). TARP develops tissue microarrays (TMA) from paraffin embedded tumoral tissues collected by the Cooperative Human Tissue Network (CHTN). Several studies have made use of these TMAs to analyze the expression of various tumor-associated markers^9–15^.

Racial/ethnic differences have been observed in cancer biomarker levels^16,17^. For instance, Preat *et al*.^16^ showed that Ki-67 labeling index, a biomarker of invasiveness in breast cancer, was higher in Arab/Moroccan patients compared with European individuals^16^. Similarly, Yamoah *et al*.^17^ found differential expression of six prostate cancer-associated biomarkers (AMACR, ERG, SPINK1, NKX3-1, GOLM1 and AR) between African American and European American patients. These markers predicted the risk of clinic-pathologic outcomes in an ethnicity-dependent manner^17^.

The advances in “omics” technologies have led to an improved understanding of cancer. Cancer multi-omics has facilitated the molecular characterization of a wide range of human cancers. For instance, The Cancer Genome Atlas (TCGA) and Therapeutically Applicable Research To Generate Effective Treatments (TARGET) efforts aim to identify molecular alterations, at the protein, RNA, DNA and epigenetic levels, to establish tumor classifications with improved accuracy^3^. These genomic signatures allow clinicians to administer personalized cancer treatments^13,18–21^. Similarly, The OncoArray Consortium aims to lay the genetic groundwork of breast, ovarian, prostate, colorectal, and lung cancers^22^. Additionally, cancer-related genome-wide association studies (GWAS)^23^ are also improving our understanding of cancer biology^24^. However, Popejoy & Fullerton^25^ showed by analyzing 2,511 GWAS (35 million samples) that the majority of these samples (81%) were isolated from European descents^25^. These data were obtained using the publicly available GWAS catalog; however, analysis focused on cancer GWAS studies has never been performed.

Basic oncological research aims to identify the underlying biological processes involved in cancer. From such understanding, several anti-cancer drugs have been developed and tested in clinical trials. Racial/ethnic differences in drug response have also been reported in many studies^26,27^. Among the most prominent examples of race/ethnicity-based drug response is found in Asian populations with lung adenocarcinoma (AD). Chinese, Korean, and Japanese female non-smokers with AD presented a higher prevalence of mutations in the epidermal growth factor receptor (EGFR) gene compared to Whites.

Patients with these alterations, which are clustered between exons 18 and 21 of EGFR, respond very well to EGFR tyrosine kinase inhibitors, such as erlotinib or gefitinib^28,29^. In addition, clinical trials of bevacizumab (to treat stomach cancer) and cetuximab (to treat non-small cell lung cancer) have shown effectiveness only in White patients^27^. Also, genetic-based pharmacoethnic differences in drug pharmacokinetics and pharmacodynamics have been reported for many compounds^30–34^.

In addition, a series of reports have shown that race/ethnicity has a great impact on cancer incidence^26,35–37^, survival^38–41^, drug response^42,43^ cancer molecular pathways^44–46^ and even on cancer-related epigenetic phenomena^47–49^. For instance, a lower incidence of glioma has constantly been reported in African Americans compared to Caucasians: 3.6 vs. 6.7 per 100,000 adults^50,51^. Additionally, it is largely known that non-Hispanic Black women and Hispanic women with breast cancer have a higher risk of cancer mortality compared to non-Hispanic White women^52^.

Concerning ethnic differences in cancer molecular mechanisms, a well-reported example is found in the TP53-mediated apoptosis pathway. TP53 is a well-known tumour suppressor that controls growth arrest and apoptosis. Chen *et al*.^53^ showed that non-Caucasian adult patients with glioma were five times more likely to present TP53 mutations in exons 5–8 compared with other populations^53^. Concomitantly, Hill & Sommer^54^ reported that TP53 mutational pattern differs among 15 geographically and ethnically populations^54^.

Regarding cancer epigenetics, ethnic differences in DNA methylation patterns have been described in several cancer types (e.g. lung, prostate, breast and colorectal)^47–49^. For instance, in lung squamous cell carcinomas (SCCs), Piyathilake *et al*.^55^ suggested that DNA hypomethylation is involved in the progression of SCCs in Caucasians but not in African Americans^55^.

Although the reasons underlying these observations have not been fully understood, it seems that differences in tumor genomics, health care access, disease detection, quality of treatment and lack of participation in health research may contribute to these outcomes^17,26,50,56–64^. For example, it has been estimated that approximately 1 per each 20 adult cancer patients participate in clinical trials; this rate persist over the time^65^ and is significant lower among racial/ethnic minority groups^57,65^. Despite several U.S initiatives lead by the Federal Drug Administration (FDA), the National Institutes of Health (NIH) and the Centers for Medicare and Medicaid Services, minorities are still underrepresented in clinical trials^56^.

Along with many other factors, racial/ethnic disparities produce inequalities in health care quality^66–68^. Eliminating these disparities is of a great interest because population demographics are constantly evolving and medical expenditures could be reduced^66–68^. For example, it is projected that by 2050 the majority group will no longer be non-Hispanic whites in the United States^69^. Additionally, it is estimated that the U.S. cost of this disparity to be in excess of $245 billion dollars annually^70,71^. Despite the clear influence of race/ethnicity in cancer outcomes and its impact in health care quality, an overview of racial/ethnic inclusion in oncological research has not been addressed yet. To have a complete understanding of racial/ethnic inclusion in cancer research, we have studied these demographic characteristics in several aspects of oncological research, from cell lines and patient-derived xenografts to biobanking, genomics and clinical trials.

## Results

### Fundamental cancer research

#### Patient-derived oncological models

**Figure 1:**
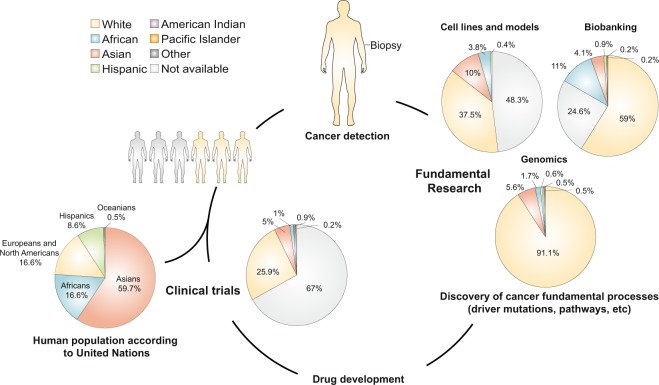
Racial/Ethnic disparities in cancer research. Racial/ethnic inclusion was studied in several aspects of oncological research, from cell lines and patient-derived xenografts to biobanking, genomics and clinical trials.

Racial/ethnic status was collected from the most common cancer cell lines and tumor samples available at the NCI PDMR. The majority of cancer cell lines (n = 689) have no records on race/ethnicity (46.1%). The rest were isolated manly from Whites (37.7%), followed by Asians (11.6%), African Americans (4.2%) and Hispanics (0.4%). The same tendency is observed in tumor samples available at the PDMR (n = 105). We found no racial/ethnic data for the majority of samples (62.86%). Of those with race/ethnicity reported, 36.19% were obtained from Whites and 0.95% were obtained from African Americans. Overall (n = 794), we found that 48.3% of samples have no records on their donor’s race/ethnicity. The remaining specimens were isolated mainly from White patients (37.5%), followed by Asian (10%), African American (3.8%) and Hispanic (0.4%) donors (Fig. 1). Supplementary tables 1 and 2 have detailed information of both datasets.

#### Biobanks

Race/ethnicity information was collected from TARP repository, the Penn-CHOP Tumor Tissue Bank, the Children’s Brain Tumor Tissue Consortium (CBTTC) and the Komen Tissue Bank. Overall (n = 8,293), no data on race/ethnicity was found in 24.56% of samples. The rest were isolated from White people (59.03%), followed by African Americans (11.05%), Asian/Pacific Islanders (4.29%), Hispanics (0.87%) and American Indians (0.2%). Supplementary Table 3 contains detailed racial/ethnic information of all biobanks analyzed.

#### Cancer genomics

We collected racial/ethnic records from four major cancer genomic projects: TCGA, TARGET, cancer-related GWAS and OncoArray Consortium. NCI TCGA and TARGET projects (n = 12,980) did not report racial/ethnic data on 11.6% of their individuals. Specimens were donated mainly by White individuals (73.3%), followed by African American (8.9%), Asian (5.6%) and other donors (0.6%). Supplementary Table 4 contains detailed racial/ethnic information of all cancer types studied by TCGA and TARGET projects.

Racial/ethnic registers of 416 cancer-related GWAS (n = 6,375,784) showed that 0.64% of individuals have no data on race/ethnicity. The majority of individuals are Whites/Europeans (91.56%), followed by Asians (5.45%) and Hispanics (0.55%). Other race/ethnicities were represented by 0.33%. Supplementary Table 5 comprises detailed racial/ethnic information of this dataset. Additionally, race/ethnic information of the OncoArray Consortium (n = 314,268) was reported as follows: Whites (83.43%), Asians (8.97%), African Americans (4.59%) and other races/ethnicities (3.01%) (Supplementary Table 6).

*In toto* (n = 6,765,447), no racial/ethnic data was reported in 0.6% of individuals. Tumor samples were collected predominantly from Whites (91.1%), followed by Asians (5.6%), African Americans (1.7%), Hispanics (0.5%) and other populations (0,5%) (Fig. 1).

### Applied cancer research

#### Clinical trials

We studied the racial/ethnic status of all patients involved in clinical trials of melanoma (2015 to 2017, n = 15,356), breast (2016 to 2017, n = 60,746) and lung cancer (2016 to 2017, n = 13,110). Overall (n = 89,212), no racial/ethnic registries were found in 66.95% of patients. Records were mainly obtained from Whites (25.94%), followed by Asians (4.97%), African Americans (1.08%), Hispanics (0.16%) and other minorities (0.9%). Supplementary Tables 7–9 contain detailed information of clinical trials analyzed in this study.

## Discussion and Future Perspectives

On the basis of these select studies, we observe that some aspects of basic cancer research (patient-derived models, biobanks and genomics) and clinical trials have failed to record and/or report racial/ethnic information, as well as to include ethnically diverse populations. Thus, our analysis of racial/ethnic representation in select basic and applied cancer research studies revealed two tendencies: lack of racial/ethnic information and an overrepresentation of Caucasian/White samples/individuals.

Basic cancer understanding and initial drug screening have been accomplished using cell lines isolated mainly from White and unknown patients. For instance, the majority of the NCI-60 panel (33 out 60) have no records on their donor’s ethnicity, a tendency also observed throughout the entire dataset. Importantly, these 689 cell lines are the most studied in cancer research^72^ and constitute a representative sample (64.39%) of known cancer cell lines catalogued to date (n = 1070)^1^. Despite the proven importance of racial/ethnic inclusion in cancer research, these observations persist in modern patient-derived oncological models available at the PDMR^6^. In addition, a recent report showing the development of a comprehensive melanoma PDX collection does not provide any racial/ethnic information^73^, and this data is also missing from the Cancer Cell Line Encyclopedia (CCLE)^1^ and the Genomics of Drug Sensitivity in Cancer (GDSC) cell line collection^74^.

Similarly, a significant proportion of specimens available at biobanks lack racial/ethnic information or were isolated from White individuals. Since only biobanks that provide public data access were selected for this study, biorepositories were reduced to those present only in United Sates.

Racial/ethnic registers of these U.S. biospecimens may reflect therefore the U.S. population (White 73.60%, African American 12.60%, Asian 5.10% and Other 8.7%)^75^ and not an overrepresentation of White individuals; nonetheless, a significant proportion of these samples (24.56%) lack racial/ethnic information. Racial/ethnic registers of these U.S. biospecimens should reflect the U.S. population broadly (White 73.60%, African American 12.60%, Asian 5.10% and Other 8.7%)^75^ and not over represent Whites.

These tendencies persist in two major genomic efforts to understand the molecular basis of cancer: TCGA and TARGET projects, which are vastly used by medical and non-medical scientific communities. Also, cancer-related GWAS and OncoArray Consortium database are overrepresented by White/European-descendant populations. Interestingly, these results differ from the world population^76^: Asians represent 59.7%, Africans 16.6%, Europeans and North Americans 14.6%, Hispanics (Latin America and the Caribbean) 8.6% and Oceanians 0.5% (Fig. 1.). Since these international projects include cancer samples from all over the world, no limitations were found to globally address racial/ethnic status in cancer genomics. For example, the 416 cancer GWAS^23^ include genomic projects from China, India, Japan, Canada, among others. Similarly, the OncoArray Consortium is formed by a network including several European countries, the United States, Australia, China, Korea and Canada^22^. With more than six million individuals studied, we consider that these databases^3,22,23^ vastly represent cancer genomics globally.

Concerning clinical trials of melanoma, lung and breast cancer, racial/ethnic information is frequently unreported despite the fact that genetic-based pharmacoethnic differences in drug response have been well documented^30–34,77^. This raises serious concerns for future cancer clinical and drug development guidelines. Lung and breast cancer were selected for this study because they are diagnosed with the greatest frequency worldwide^78^. Similarly, melanoma is the most commonly diagnosed cancer in western countries and its treatment changed importantly when BRAF/MEK inhibitors and immunotherapy became the new standard therapy^79^. However, more research is needed to globally address racial/ethnic status in all cancer types.

Recent comprehensive analyses have provided a solid groundwork of human genetic variation that may possibly contribute to the race/ethnicity-related differences observed in cancer outcomes^80–82^. The 1000 Genomes Project Consortium has analyzed 2,504 genomes of different ancestry (26.4% African, 20.1% East Asian, 20.1% European, 19.5% South Asian and 13,9% Latin American) across five continental regions. This consortium identified a massive number of 88 million variants among 26 human populations^81^. Similarly, the Exome Aggregation Consortium (ExAC), analyzing 60,706 exomes of diverse ancestries (60.4% European, 13.6% South Asian, 9.5% Latin American, 8.6% African, 7.1% East Asian and 0.7% Other) has identified 7.4 million variants^82^. These results underscore the relevance of considering racial/ethnic-based human genetic variation as a critical factor in oncological research^83^.

Some initiatives have taken place over the last years to increase underrepresented minorities in cancer research^84^. For instance, the Hoy y Mañana project aims to increase biospecimen donation of ethnically diverse populations^8^. Similarly, the Geographic Management Program (GMaP) and the Minority Biospecimen/Biobanking - Geographic Management Program (BMaP) aim to reduce cancer related racial disparities by implementing a multi-institutional network infrastructure in the United States^85^. In this regard, BMaP for region 3 (Southeastern United States and Puerto Rico) developed and validated TMAs of invasive ductal carcinoma from ethnically diverse populations^86^. In addition, the U.S-based National Institute on Minority Health and Health Disparities (NIMHD) leads scientific research to reduce health disparities and improve minority health focusing on cardiovascular diseases, diabetes and cancer. Also, several studies have analyzed cancer-related genes of underrepresented human populations, such as Native Americans and Mestizo populations^87–91^.

Samples collected by the aforementioned strategies should be predominantly included in basic aspects of oncological research, such as patient-derived oncological models, initial drug screening and cancer genomics. This will alleviate racial/ethnic disparities in fundamental cancer research and further drug development. This should be enhanced by legal regulations in health policies. For example, the NIH Revitalization Act of 1993 should establish inclusion of minorities not only in clinical trials but also in fundamental cancer research, such as development of patient-derived cancer models (PDXs and PDCs), biobanks and genomics. Also, other legal initiatives should endorse race/ethnicity recording and reporting in all aspects of fundamental and applied oncological research.

To improve minority representation in cancer research, research agencies worldwide should promote fundamental projects to develop patient-derived models, biobanks and cancer genomics projects based on their populations. Also, clinical trials of new anti-cancer drugs should be extended to other countries and supported by international collaborations. Racial/ethnical disparities could also be reduced by increasing the participation of minorities in research projects.

In this concern, several studies have been performed and many strategies have been suggested to increase participation of underrepresented populations^56,57,63,64^.

Additionally, race/ethnicity should be determined by more accurate approaches such as genetic-based ancestry identification methods; for instance, race/ethnicity of genomic samples could be determined *in silico* using ancestry markers^92^.

## Methods

### Racial/ethnic categories

Since many studies analyzed in this work were performed by U.S. initiatives, we decided to standardized our data using the U.S. federal register 62 FR 58782^93^ to classify race and ethnicity.

### Cancer cell lines and modern oncological models

Cell lines dataset were constructed as follow: the NCI-60 panel was merged with the 675 most frequently used cancer cell lines^72^, giving a total number of 689 cell lines. Racial/ethnic information was obtained from Klijn *et al*.^72^ and updated using: ExPASy-Cellosaurus (https://web.expasy.org/cellosaurus/), COSMIC Cell Lines Project v83 (http://cancer.sanger.ac.uk/cell_lines), HyperCLDB^94^ (http://bioinformatics.hsanmartino.it/hypercldb/indexes.html), the catalogue of Deutsche Sammlung von Mikroorganismen und Zellkulturen (DSMZ) human and animal cell lines (https://www.dsmz.de/catalogues/catalogue-human-and-animal-cell-lines.html), European Collection of Authenticated Cell Cultures (ECACC) (https://www.phe-culturecollections.org.uk/collections/ecacc.aspx) and the Japanese Collection of Research Bioresources Cell Bank (JCRB) (http://cellbank.nibiohn.go.jp/). To increase our analysis on patient-derived oncological models, we include the NCI PDMR, which represents an improved version of the NCI-60 panel^95^. Racial/ethnic registries from PDMR (n = 105) was obtained from https://pdmr.cancer.gov/.

### Biobanks

We initially consider 15 international cancer biobanks^96^ in addition to 3 U.S. biorepositories and 1 published data from the Komen Tissue Bank^97^ (also from U.S.). Since only biobanks that provide public data access^96^ were selected for this study, 15 biobanks were excluded from our analysis: Australasian Biospecimen Network (Australia), Australian Prostate Cancer BioResource, BancoADN (Spain), Canadian Tumor Repository Network, Centro National de Investigationes Oncologicas Tumor Bank Network (Spain), Chernobyl Thyroid Tissue Bank (Russian Federation), Confederation of Cancer Biobanks (UK), Cooperative Human Tissue Network (USA), Kathleen Cuningham Consortium for Research into Familial Breast Cancer (kConfab; Australia), onCore UK (UK), Singapore Tissue Network, European Human Tumor Frozen Tissue Bank, UK Biobank, Victorian Cancer Research Tissue Bank (Australia) and Wales Cancer Bank. Thus, we studied race/ethnicity of all samples available at the TARP repository (n = 1,203), the Penn-CHOP Tumor Tissue Bank (n = 1,815) and the Children’s Brain Tumor Tissue Consortium (CBTTC) (n = 2,302). We also included recent data from the Komen Tissue Bank (n = 2,973), which harbors normal breast tissue for cancer research^97^. Racial/ethnic data from TARP biorepository was obtained at https://ccrod.cancer.gov/confluence/display/CCRTARP/Home. Racial/ethnic information of The Penn-CHOP Tumor Tissue Bank and the CBTTC were obtained through the Biorepository Portal Toolkit^2^.

### Genomics

We initially selected 5 major cancer genomics projects: 1) TCGA^3^, 2) TARGET (https://ocg.cancer.gov/programs/target), 3) GWAS related to cancer^23^, 4) OncoArray Consortium^22^ and 5) The Chinese Cancer Genome Consortium (CCGC)^98^. To our knowledge, these projects vastly represent cancer genomics globally^3,22,23^. CCGC (n = 260) was excluded from our analysis because the raw data is still not publicly available. Thus, demographic characteristics (race and ethnicity data) were obtained from 33 TCGA projects and 6 TARGET projects (Supplementary Table 4) through the NCI’s Genomic Data Commons (GDC, https://gdc.cancer.gov/). 416 cancer-related GWAS^23^ (Supplementary Table 5) were selected from the GWAS catalog comprising 6,375,784 samples (https://www.ebi.ac.uk/gwas/). Racial/ethnic information was obtained from the OncoArray Consortium database^22^ (n = 314,268) through https://epi.grants.cancer.gov/oncoarray/ (Supplementary Table 6).

### Clinical trials

All clinical trials (randomized or not) were systematically selected from PubMed, associated with lung (from December 2016 to December 2017, n = 13,110 participants), breast cancer (from December 2016 to December 2017, n = 60,746 participants) and melanoma (from January 2015 to March 2017, n = 15,356 participants), which are related with active treatments (oncospecific drugs, radiotherapy and surgery). Racial/ethnic information was obtained from 55 studies in melanoma, 71 in breast cancer and 82 in lung cancer (Supplementary Tables 7–9).

## Electronic supplementary material

Supplementary Dataset 1
